# Teaching public health in undergraduate medical courses: a case study in three universities in Paraná

**DOI:** 10.1590/S1516-31802009000600003

**Published:** 2010-05-21

**Authors:** João José Batista de Campos, Paulo Eduardo Mangeon Elias, Luiz Cordoni

**Affiliations:** I MD, PhD. Professor in the Department of Public Health, Universidade Estadual de Londrina (UEL), Londrina, Paraná, Brazil.; II MD, PhD. Professor in the Department of Preventive Medicine, Faculdade de Medicina da Universidade de São Paulo (FMUSP), São Paulo, Brazil.; III MD, PhD. Senior Professor in the Department of Public Health, Universidade Estadual de Londrina (UEL), Londrina, Paraná, Brazil.

**Keywords:** Curriculum, Education, medical, Public health, Education, medical, undergraduate, Education, higher, Currículo, Educação médica, Saúde pública, Educação de graduação em medicina, Educação superior

## Abstract

**CONTEXT AND OBJECTIVE:**

Historically, different concepts of public health have influenced both the specific teaching in this field and its participation in general physician training. Starting from this assumption, the objective of this paper was to study how public health has been taught in undergraduate medical courses, focusing on structure and on how this has affected curriculum design in three universities in the State of Paraná, Brazil.

**DESIGN AND SETTING:**

Qualitative investigation developed at Universidade Federal do Paraná (UFPR), Universidade Estadual de Londrina (UEL) and Universidade Positivo (UnicenP).

**METHODS:**

This study included a documentary analysis on pedagogical projects and on how these are actually experienced by those working on them. Eleven managers and 18 teachers were interviewed, as well as four groups of students that were formed in the three medical courses.

**RESULTS:**

Between 5 and 20% of Public Health topics were shown to be included in the curriculums, depending on the teaching strategies used. However, they were always set up within academic approaches that were strongly linked to healthcare services. This situation has been strengthened through the degree of progress made by the National Health System (Sistema Único de Saúde, SUS) in both cities (Curitiba and Londrina).

**CONCLUSIONS:**

Regardless of the nature of the university, the administrative and academic setup of the course and of the different ways of incorporating teachers, Public Health is present and takes on considerable relevance for medical training, even if it does not constitute a linking thread within undergraduate medical courses.

## RESUMO

**CONTEXTO E OBJETIVO:**

Historicamente, as diferentes concepções de saúde coletiva influenciaram tanto no ensino específico desse campo do saber como na formação geral do médico. Partindo desse pressuposto, o trabalho teve como objetivo estudar o ensino da saúde coletiva na graduação médica, buscando apreender suas configurações e as implicações delas nas propostas curriculares de três universidades do Paraná (Brasil).

**TIPO DE ESTUDO E LOCAL:**

Pesquisa qualitativa desenvolvida na Universidade Federal do Paraná (UFPR), Universidade Estadual de Londrina (UEL) e Universidade Positivo (UnicenP).

**MÉTODOS:**

Este estudo incluiu uma análise documental dos projetos pedagógicos e de como estes são na realidade vivida por aqueles que trabalham com eles. Onze gestores e 18 professores foram entrevistados, bem como quatro grupos de estudantes, que se reuniram nos três cursos de medicina.

**RESULTADOS:**

Evidenciaram a inserção de 5% a 20% dos temas da Saúde Coletiva nos currículos, dependendo das estratégias de ensino, mas configurando-se sempre em abordagens acadêmicas fortemente vinculadas aos serviços de saúde, uma realidade que é fortalecida pelo grau de adiantamento do Sistema Único de Saúde em ambas as cidades (Curitiba e Londrina).

**CONCLUSÕES:**

Independentemente da natureza da universidade, da configuração burocrática e acadêmica do curso e dos diferentes modos de inserção docente, a Saúde Coletiva se faz presente e se assume com considerável relevância para a formação médica, ainda que não se constitua um eixo articulador da graduação em Medicina.

## INTRODUCTION

Public health has been defined as a field of knowledge and practice for which it is assumed, as a prerequisite, that health is understood as an eminently social and public phenomenon that is historically determined by the living conditions and lifestyles of different population groups. The epistemological and theoretical debate that exists today within the field of Public Health provides a more advanced concept of health, as a subject of knowledge and action, and it is understood as part of the “health-disease-care” complex. It also incorporates historical rapports that determine public health, including individuals’ relationships and social groups as they relate to healthcare services.^1^^,^^2^^,^^3^

The emphasis on these key public health concepts does not exclude factors that lead to this central understanding. These determinants include the current national historical moment and health map, along with the attitudes of physicians who are currently undergoing training, towards our country’s health policies. Hence, this study focused on the impact of many different understandings of public health and how they affect both the field of public health and physicians’ education.

The questions that arise are the following: How should academic education be understood? How should physicians be educated? What is the best method to teach medicine? Lastly, why is it important to work on this issue, and why is this justifiable?

In the field of education, three major educational models or trends that generally guide pedagogical projects, educational practices and even the relationship between teachers and students can be identified. These are empiricism, innatism (or the innate, spontaneous, improvised approach) and constructivism.^4^^,^^5^^,^^6^

Another assumption of this study is that that the physicians to be produced are not people who know a bit of everything, but professionals who will be educated to acquire broad and scientifically based medical knowledge. This will enable them to attain a good level of professional performance, which will not require an early specialization process, as if just “minimal knowledge” were sought. Physicians of the future must be capable of seeing their profession in its real sociopolitical context. They should be professionally inquisitive, investigative and critical, and able to function in the real healthcare world and within their surrounding context.^7^^,^^8^^,^^9^^,^^10^

This study was undertaken in order to understand different public health trends. These need to be considered not only because of their relevance to established undergraduate medical courses, but also because of how they affect all the players involved: administrators, teachers and students.

Furthermore, this contextual knowledge of Public Health and its role in medical education seems particularly relevant within the Brazilian political and academic context, in which national curricular guidelines for undergraduate medical courses are under discussion. This approach is consistent with the principles and innovations of the Brazilian National Health System (Sistema Único de Saúde, SUS), given that even 20 years after its establishment, an old discussion between the favoring of technical excellence versus the favoring of social relevance continues to reverberate.^11^^,^^12^

## OBJECTIVES

This paper aimed to study how Public Health has become part of undergraduate medical courses, by picking out its tenets and the implications of these proposals in the curriculums of three universities in the State of Paraná, Brazil: Universidade Estadual de Londrina (UEL), Universidade Federal do Paraná (UFPR) and Universidade Positivo (UnicenP).

The specific objectives of this study were to describe the public health educational projects in three medical schools in this State (UEL, UFPR and UnicenP) and to compare them with each other and in relation to the new national guidelines for the field of healthcare. This study also sought to understand and analyze the public health skills that physicians should have by the time they graduate. These skills were compared with adequate productive levels within healthcare. The ways in which public health has contributed towards the innovations in the national syllabus innovations, and how these are relevant in developing undergraduate medical courses, were reviewed.

## METHODS

This study was developed from three case studies that were based on analysis of documents produced by these medical schools and on interviews with administrators, teachers and groups of medical students. All of these players are considered important with regard to understanding the educational process surrounding the introduction of public health at the undergraduate level in the institutions examined. The methodological choice of studying three cases was based on the idea that by closely examining a particular group or unit, its characteristics might be understood in depth.^13^

The study on each institution started by gathering documents on the programs, educational plans and syllabus guidelines, with the aim of conducting a documentary analysis on each program in the form in which it is officially established.

In addition to gathering documents, it was considered of interest to obtain interpretations and perceptions from people involved in these programs. This would make it possible to pick up what is actually experienced and generally understood regarding daily life in each of the institutions.

In studying the pedagogical projects of these three institutions, we also identified the educational trends in public health. The idea of performing content analysis on the interviews, with consideration of their points of agreement and disagreement, had the aim of showing what was studied and how educational proposals can be implemented in different ways. The aim here went beyond simply comparing different alternatives for Public Health teaching: the objective was to capture what the educational leaders considered important in their projects.

This is why we chose to hold interviews with representatives from each institution. While guided by these objectives and identifying equivalent points, the actual interview process was adapted to each group of individuals: managers, teachers or students.

For the students, the technique used was focus groups: these used guided questions, including students’ self-perceptions regarding various public health educational activities that were developed during their medical courses.

In the documentary analysis on the institutions, after exhaustive reading of the main legal texts relating to the pedagogical project and, more specifically, texts about how Public Health is taught, the university’s most important resolutions were identified. Each case was handled carefully with regard to the diversity of documents and the nature of each university’s higher councils and administrative systems.

In turn, the analyses on the data collected during the interviews and from the focus groups was guided by establishing categories that emerged from the content analysis and by mapping out and organizing the units of meaning. According to how peoples’ opinions were considered, different aspects of public health could be established and compared: planning, experiences and syllabus analysis.

These semistructured interviews were prepared and implemented by a single interviewer, who conducted them orally, with recordings, and then transcribed them. This made it possible to appraise the data using Bardin’s method for content analysis.^14^ This method consists of extracting and sorting the content identified from semistructured interviews. The content was put into conceptual categories according to the emphasis considered, in order to understand the educational approaches selected.

The content that emerged from the analysis on the interviews conducted at the three institutions were systematized into three main categories: 1) organization of Public Health; 2) Public Health within the context of the medical course, and, 3) external Public Health partnerships. This served as the reference frame for the entire categorization analysis.

This investigation was approved by the Ethics Committee that reviews research projects (Comissão de Ética para Análise de Projetos de Pesquisa, CAPPesq) at Hospital das Clínicas (HC) and at Faculdade de Medicina da Universidade de São Paulo (FMUSP). The interviewees (managers, teachers and students) signed a free and informed consent statement, after receiving explanations about the general objectives of the study. Assurances were given regarding the confidentiality of the information provided.

## RESULTS

This study was carried out in 2007 in three universities in the State of Paraná, Brazil. The population involved was formed by 11 managers, 18 teachers and 29 students of the medical course of these universities. There were significant differences along three notional axes between the three institutions examined, both in relation to the secondary data from the documents gathered and in relation to the different points of view among the individuals interviewed at UEL, UFPR and UnicenP.

### UEL medical course

Faculdade de Medicina do Norte do Paraná (FESULON) began its academic activities on February 15, 1967. In 1970, when UEL was inaugurated, the medical course became part of the Health Sciences Center (Centro de Ciências da Saúde, CCS).

Over the first 40 years of existence of the medical course, it was observed, that on average, the medical course guidelines changed every six years. Such occurrences are uncommon among medical schools, which generally suffer from having rather rigid programs. Over the past ten years, an integrated program has been created.^15^

This brief history characterizes a medical course that is committed towards new academic models, thus resulting in its unconventional curriculum matrix. Information is available about who coordinates curricular years and modules, and on which days activities are developed, other than small-group tutorials. These activities may relate to clinical skills, with assessments made using objective, structured clinical examinations (OSCEs).^*^

Over the years, UEL’s Public Health teaching staff have established a consensus regarding their contribution towards the transformation process of the educational institution. This includes healthcare innovations, considering that changes in organization, practices and results will build a fairer and more inclusive social system.

Since 1998, in the new integrated Medicine syllabus, the teachers of the Department of Public Health have started to act more intensively in four modules that together represent 5% of the total timetabled hours of the program.

### UFPR medical course

The School of Medicine was founded in 1912. The Medical School program is administered by the UFPR Division of Health Sciences, which was formed in 1972. At that time, new facilities were inaugurated. They are attached to the Hospital de Clínicas, in Curitiba.

The Medical School program is structured into academic subjects and the academic regime is based on credits. The Department of Community Health at UFPR was originally formed through merging two chairs in the medical course: Tropical Diseases and Hygiene.

Since 1994, with the implementation of a reform of the Community Health program, teaching staff have begun to work more regularly on seven subjects in this program. In terms of teaching hours, this program represents 6% of the total. However, after adding the internship rotation, the number of hours increased to 12% and this addition became mandatory once the medical course was established in today’s institution.

The qualitative impact of this adjustment on the evaluation process for the UFPR Medical School remains an open question, to be answered by the program administrators once the transition program has been completed (2012), at which time the school will be 100 years old.

### UnicenP medical course

The UnicenP medical school was formally established in 2002 and the medical course was recognized by the Ministry of Education in 2008. The first class began its academic activities in March 2003 and graduated at the end of 2008.

According to their pedagogical project, the medical course was structured as a family health program, which is one of the concepts strongly worked up by the program. The objective was to train qualified, good general physicians.

The field of Public Health, like some others in this university, has not been established as a Department. It is forms part of the Biological and Health Sciences unit, which brings together the teachers of all eight of the institution’s health programs.

The coordination for this unit and the medical course is interlinked, and the latter manages both academic and administrative matters. Teachers are recruited according to the number of teaching hours. Public Health teachers represent 20% of the staff, and this total, comprising 64 teachers, is directly responsible for the program results.

The teaching hours within Public Health represents 20% of the total teaching hours of the program, distributed across six subjects. Out of this total, 7% are developed between the first and fourth years and 13% during medical internship, which reflects the concern for being in consonance with the recommendations of the National Curriculum Guidelines for Undergraduate Medical Programs, as approved by the Ministry of Education (MEC).

## DISCUSSION

According to Gil,^13^ analysis on units within a given universe meaningfully facilitates general understanding of this universe. Case studies consist of detailed examination of cases with unique characteristics, within a real context, using multiple sources.

Together, these institutions represent 49% of the first-year medical course places in Paraná, which also ensures a good sample of the students who enrolled in 2006. In addition, the syllabus of UEL is considered to be the most innovative among the universities in Paraná,^16^ while UFPR has the most traditional syllabus of all and UnicenP, the newest of these institutions, is the only private institution in the state.

In this sense, there is an intricate relationship between breadth and depth in qualitative studies, and difficulties in conducting studies in greater depth arise if the magnitude of such studies is too large.^17^ This is the reason why the study on these three programs was limited.

In Paraná in 2006, there were seven medical schools. Nationwide, 132 medical schools were members of the Brazilian Association for Medical Education (Associação Brasileira de Educação Médica, ABEM), of which 52 were public and 80 were private.^18^

According to Yin,^19^ the multiple possibilities of case studies reflect the diversity of how searches may be conducted. On the other hand, it is also important for researchers, in choosing this method to answer questions in surveys, to be sure of its appropriateness. For this method to be undertaken, there need to be well-defined criteria for case selection.^20^

In our case, however, this selection had the merit of ensuring representativeness, through incorporating the three legal types of higher education institution in the state of Paraná: UEL (state and public), UFPR (federal and public) and UnicenP (private).

Despite the different curriculum configurations, great convergence of opinions was observed in all four student focus groups in the three institutions. What varied between the three cases was the teaching hours and the emphasis attributed to each Public Health program. These programs could be organized as modules, selections of subjects, apprenticeships or practice sessions, in addition to the theory load established by each program.

### The curriculum and SUS

Views about the National Health System (SUS)^21^ and the concepts behind its health service provision only appeared explicitly in the students’ focus group, among UEL interviewees. At UFPR, other than the students, only one teacher also made reference to this subject. At UnicenP, there was considerable debate in the students’ focus group. One teacher and one administrator commented on this subject.

Differences among students’ and teachers’ perspectives relating to SUS and its principles, despite the 20 years for which it has now been legally constituted, might be more directly related to the social origins of the individuals concerned. This might be explained if these views are considered as “popular knowledge and common sense”, elaborated and shared publicly, as a means of constructing and interpreting reality.^22^

It was noticeable that the meanings and values that the students ascribed to SUS were quite different from those of their teachers. This shows that several forms of conceptualization existed, according to the individuals’ knowledge or lack of knowledge about city-level health service organization, financial difficulties, policies and management decentralization.^23^

It is worth emphasizing one common point among the institutions studied: these medical courses are in two cities where SUS implementation is very advanced. Thus, regardless of whether the teachers are at institutions with participation in research, or are only employed on an hourly basis, these characteristics seem to have little significance for differentiating teachers with regard to medical education if their viewpoints are considered.

### What has been done and what needs to be done in Public Health

Given the different profiles of medical education and the respective commitments to train capable professionals to work towards the challenge of implementing SUS, the responses to our survey provide a real statement of what has been done and what needs to be done.^24^

Thus, the various views of what Public Health is might help to interpret the meanings and challenges envisaged by the interviewees at UEL, UFPR and UnicenP.

In the case of UEL, even before the promulgation of the new national constitution in 1988 brought SUS into existence, the Medical School had already pioneered an agreement with the Municipal Health Department to establish a Health Center at Villa da Fraternidade in 1970. This center is committed towards building favorable scenarios for primary healthcare education in Londrina.^25^

In Curitiba, the capital of the state of Paraná, this process happened after the establishment of SUS and had a positive influence on the UFPR Medical School. This included the introduction of general clinical practice in the curriculum, in 1994.^*^

Within the UnicenP medical course, there has been an attempt to build a channel towards Family Health, in accordance with national guidelines. This program is being implemented in the Santa Felicidade district of the city of Curitiba.

The Public Health initiatives and achievements within the UEL medical course and as exemplified by regional and national health policies have always been directed towards fostering change in medical education. In addition to some emblematic historical figures among hygienists who have worked in Londrina, other teachers over the past 40 years have also played outstanding roles in formulating and interconnecting the transformational movement of the program and in developing human healthcare resources.^26^

Within Public Health at UEL, whether at undergraduate or postgraduate level, there has always been concern for educational issues relating to medical training.^27^ This is shown by the creation of a workstation at the Public Health Study Unit (Nesco) of UEL, which has formed part of the Observational Network for Human Healthcare Resources (Ministry of Health/Pan-American Health Organization, PAHO) since 1999.^28^

The way in which one of the most important local organizational processes for healthcare services was built up in Curitiba is well described in two special issues of *Divulgação,* a magazine for healthcare debate published by the Brazilian Center for Healthcare Studies (Centro Brasileiro de Estudos de Saúde, CEBES): issue no. 8 (Health and Change, 1992) and issue no. 19 (Curitiba: 20 years of Primary Health Care, 2000). The content of these articles was compiled under the responsibility of Curitiba’s Municipal Health Department.

In issue no. 8 of this magazine, Raggio and Romanó Júnior^29^ open the profile section with a proposal to produce community general practitioners to work in Curitiba’s Health Centers. Other sections of this issue contained opinions, interviews, care models, public actions, new attitudes and important issues at that time, including healthcare regionalization, with an article written by Giacomini.^30^

In the same issue of this magazine, in the article Health and Change, the Health Secretary of Curitiba, Armando Raggio, described how changes in the Municipal Health Department had been guided and directed. This provided an important contribution towards the debate at the Ninth National Healthcare Conference, in which the central theme was the municipalization of the healthcare system.

In issue no. 19 of this magazine, the article by Pedotti and Moyses^31^ analyzes the 20-year history of primary healthcare in Curitiba, starting from the Alma Ata conference and the adoption of primary care as a paradigm for Public Health. The authors explained the evolution of the most recent period for effective implementation of the Family Health model, which continued to grow and improve as a result of the Sixth Public Health Meeting of the Municipal Health Department. The other articles brought together in this issue were some of the studies from this event, which were chosen by the participating public at the event and were presented by the Health Secretary, Luciano Ducci.

In parallel with the significant changes happening to Curitiba’s health services, with regard not only to the quantitative extent of healthcare but also to the quality of this care, the organizational structure and goals of these health services need to be considered. Moreover, there have also been major academic changes, particularly at UFPR

In 1992, the managers of the UFPR medical course implemented a syllabus reform in which the teachers of the Department of Community Health ended up in the middle of a dispute between those who wanted to train physicians with a more generalist profile and those seeking to produce specialized physicians.

At that time, it was reported that the UFPR medical course syllabus was very specialized, with repetitive content focusing mostly on responses to chronic patients. These patients were users of the services of Curitiba’s Clinical Hospital, with demands that were more specialized, and a morbidity profile of uncommon problems that required interventions of greater complexity. The criticism that students made was that they attended patients whose diagnoses had already been established and did not know how to deal competently with cases from general outpatient clinics, i.e. with the capability to resolve such cases.

Public Health at UFPR showed its clear intentions to participate in the transformation of medical education in Brazil, from the time when its own situational diagnosis had been made. Subsequently, the National Inter-institutional Commission for Assessment of Medical Teaching (Comissão Interinstitucional Nacional de Avaliação do Ensino Médico, CINAEM)^32^ provided some guidance, in which the principles of universality, comprehensiveness and access to actions and services were defended through the logic of a National Health System. This took place in spite of internal resistance from teachers and operational difficulties in building effective partnerships with the Municipal Health Department.

Teaching staff at UnicenP do not have a departmental organization, but their representative highlighted the importance of Family Health as a guiding thread for the medical course. Family Health aims to interconnect content and practices throughout the medical course. The presence of Family Health teachers as coordinators for the medical course and for several other settings within the educational process is a sign of the institution’s appreciation and recognition of the formative role of this project.^33^

In addition to what has already been done, there is a big challenge for Public Health, since each course is experiencing very special moments. The first class of physicians graduated from UnicenP in 2008. UEL’s School of Medicine has just completed 40 years and UFPR is preparing for the celebrations of its centenary in 2012.

In this regard, it is worth stating that active educational methodologies are important, but they do not suffice and are not the main component of medical education. There is a need for new practice scenarios, for effective participation within community settings and health services. Moreover, the integration of determinants and a conceptually broad idea of healthcare acting as the axis for curriculum development are important and have strategic value. These came up in the testimonies of several administrators, teachers and students within the medical courses in Londrina and Curitiba.

In any event, there are signs that through adopting innovative educational programs, changes in the correlations among internal forces are occurring within academia and are an important ingredient for stimulating changes that should promote further structural and theoretical planning.

### The possible dialogue between the teaching trends within public health

Three notional axes emerged from the analysis of the interviews: the first axis consisted of Public Health organization, as reflected by its goals, emphases and prevalent strategies. The differences could be correlated with the specific syllabus for each institution and with the multiple interpretations and thoughts expressed by the administrators, teachers and students of the medical courses at UEL, UFPR and UnicenP.

The second axis consisted of public health within the context of each of the three courses examined, and how they integrated into the educational project and adhered to the national curriculum guidelines. We found differences directly relating to the presence or absence of support mechanisms and follow-up for the teaching staff who were involved with the pedagogical projects developed within the medical schools at UEL, UFPR and UnicenP.

The third axis consisted of the external partnerships between the Public Health divisions of UEL, UFPR and UnicenP and the Municipal Health Department of Londrina and Curitiba. Although the analyses on the interviewees’ statements identified differences in the integration mechanisms, the directions and intentions of all three institutions were convergent in relation to SUS. The interviewees placed importance on working more closely with the healthcare systems in each city.

Comparative analysis among the three institutions showed that the syllabuses planned at UEL, UFPR and UnicenP differed from what was actually implemented. However, this disparity did not go in the same direction in the three universities.

At UEL, given the reformulation and implementation since 2005 of a new Policy and Pedagogical Project, this disparity is smaller. This is expressed through a Resolution from the Teaching, Research and Extension Board (*Conselho de Ensino, Pesquisa e Extensão*, CEPE), No. 22/2005, which reflected decisive agreement with the National Curriculum Guidelines for medical school programs. The structural basis of the change had already been under development since 1998, with the implementation of an innovative syllabus that resulted from analyses and discussions within the Medical School Education Committee. In this institution, the disparity had a positive dimension, since the implementation of an integrated curriculum was inspirational and contributed significantly towards the final text of the official document for these National Curriculum Guidelines.

In UnicenP, the disparity between the planned syllabus and what was effectively implemented was intermediate in dimensions and, in a certain way, reflected the distance between intention and action. Because this is a new medical course that is still being implemented and from which the first class only graduated in 2008, it intentionally sought to conform to the recommended National Curriculum Guidelines, as a legitimate means of attaining legal program recognition from MEC.

In UFPR, the disparity between the planned syllabus and what was actually implemented was bigger and had a dimension of adjustment. This was because the current syllabus is now closer to the proposed National Curriculum Guidelines, and this has led to constant review through a “syllabus adjustment” process. This ongoing process has been construed as reinforcement of a fragmented, discipline-based and more traditionally organized syllabus, thereby rendering it less accepting of the original recommended proposal, as set forth in the Brazilian National Curriculum Guidelines.

### Conclusions and implications: invitations for reflection

At the end of this study, while the evidence showed that Public Health did not constitute the backbone of these medical courses, the relevance of Public Health to the education of these professionals was nevertheless clearly confirmed. The epistemological peculiarity of Public Health within the educational field provides an intellectual dimension, in that it pertains to universal knowledge and can thus be considered to fall within the best traditions of enlightenment.^34^ Hence, these traits strengthen the integrative building capabilities of Public Health within the process of its interactive relationships with the entire knowledge spectrum of the medical profession.

The complexity of physicians’ training, which needs to be considered from the perspective of many fields, not only requires the content material that is essential for integrated medical care in which high technical excellence must be developed, but also requires medical schools to be socially relevant. Given Brazil’s healthcare realities and the healthcare needs of its population, and the enormous challenges that we consequently face, social relevance is a must.^35^

Therefore, the discussions on medical education deserve to be reconsidered using two perspectives: the pedagogical ideals and the political realities.

An ideal educational perspective should be based on responsible understanding of what medical education should be. Learning that prepares students for a wide understanding of the health-disease process is certainly required, since highly qualified technical and specialized performance is needed. Nonetheless, health services must include careful working practices that support healthcare individually and publicly with proper integration between these two aspects.

Political realism seems to put forward some reasons for creating undergraduate Public Health courses in different Brazilian cities. This could lead to weakening of both multi-professional practice as well as loss of healthcare comprehensiveness during medical education.

Based on these political-pedagogical benchmarks, it is possible to set out some implications emerging from this study:

1. It is unviable to expect that a single medical education syllabus will lead to a single, rigidly formulated medical course that is good for all. This is true even if the syllabus is taken from the National Guidelines established by MEC, and even though these guidelines are increasingly supported by the Ministry of Health.
2. There is a need for medical education to consider all different sociocultural contexts and the uniqueness of different institutions. Particularly, there is a need to reconcile the educational process as one that must integrate technical knowledge and practical work in the real world.
3. It can be concluded that medical education cannot be ensured only through the accumulation and overlaying of information from different fields of medicine. Health professionals must also develop critical attitudes regarding social commitment and must establish broad-based culture within occupational health.
4. Programs that are more daring do not solve the problem of medical education, given the complexity of the issues involved. While adjustments to programs can be a positive factor for medical training quality, other factors interfere in this process, such as the traditional structure of internship rotations, which are generally considered by some to be the “salvation” of medical courses.
5. The evidence suggests that there are no fixed or rigid programs: their quality seems to depend on institutional flexibility to implement the proposed curriculum and evaluate the results. There is a need to diagnose possible errors and to correct problems. This, in turn, requires efficient dialogue among administrators, teachers and students. In other words, healthcare institutions are closely linked to their capacity for renewal and adjustment.

Thus, to place and give an effective role for public health within medical education, consideration must be given to academic diversity and towards establishing adequate commitment, in the light of today’s healthcare challenges. Through building professional competences that imply knowledge, skills and attitudes and, additionally, through developing democratic management approaches, institutions and healthcare systems may provide the required solutions.
